# Trastuzumab without chemotherapy in the adjuvant treatment of breast cancer: subgroup results from a large observational study

**DOI:** 10.1186/s12885-017-3857-5

**Published:** 2018-01-08

**Authors:** Peter Dall, Thorsten Koch, Thomas Göhler, Johannes Selbach, Andreas Ammon, Jochen Eggert, Nidal Gazawi, Daniela Rezek, Arthur Wischnik, Carsten Hielscher, Nicolas Schleif, Ursula Cirrincione, Axel Hinke, Gabriele Feisel-Schwickardi

**Affiliations:** 1 Department of Obstetrics and Gynaecology and Breast Cancer Center, Städtisches Klinikum Lüneburg, Bögelstraße 1, D-21339 Lüneburg, Germany; 2 Breast Center, Klinikum Nürnberg Nord, Prof.-Ernst-Nathan-Str. 1, D-90419 Nürnberg, Germany; 3 Onkozentrum Dresden/Freiberg, Leipziger Str. 118, D-01127 Dresden, Germany; 4 Oncology Practice, Altmarkt 20 - 24, D-47166 Duisburg, Germany; 5 Oncology Practice, Nikolausberger Weg 36, D-37073 Göttingen, Germany; 6 Oncology Practice, Xantener Str. 40, D-47441 Moers, Germany; 7 Gyneco-Oncology Practice, Lampestr. 1, D-04107 Leipzig, Germany; 8 Gynecology Department, Marien-Hospital, Pastor-Janßen-Str. 8-38, D-46483 Wesel, Germany; 9 Department of Gynecology, Klinikum Augsburg, Stenglinstr. 2, D-86156 Augsburg, Germany; 10 Gyneco-Oncology Practice, Große Parower Str. 47 – 53, D-18435 Stralsund, Germany; 11 Roche Pharma AG, Emil-Barell-Str. 1, D-79639 Grenzach-Wyhlen, Germany; 12 WiSP Research Institute, Karl-Benz-Str. 1, D-40764 Langenfeld, Germany; 13 Department of Obstetrics and Gynecology and Breast Cancer Center, Klinikum Kassel, Mönchebergstr. 41 – 43, D-34125 Kassel, Germany

**Keywords:** HER2-positive, Monotherapy, Overall survival, Propensity score analysis, Relapse-free survival

## Abstract

**Background:**

The topic of trastuzumab therapy without chemotherapy in early breast cancer (EBC) has been repeatedly discussed at international consensus meetings, but is compromised by the lack of solid evidence from clinical studies.

**Methods:**

An observational study database of patients with EBC receiving trastuzumab-containing (neo)adjuvant therapy was screened to identify those patients who did not receive cytostatic agents.

**Results:**

Of 3935 patients, 232 (6%) were identified who received no chemotherapy, being characterized by older age, worse performance status, and/or less aggressive histology. Relapse-free survival in this cohort was 84% (95% confidence interval [CI] 78–89%) at 3 years and 80% (95% CI 74–87%) at 5 years. However, these rates were significantly worse than those in the group of patients who received chemotherapy (hazard ratio 1.49; 95% CI 1.06–2.09; *P =* 0.022). A similar pattern was observed for overall survival, with marginally non-significant inferiority in the group receiving no chemotherapy (hazard ratio 1.56; 95% CI 1.00–2.44; *P =* 0.052). Survival rates in patients receiving no chemotherapy were 93% (95% CI 88–97%) and 87% (95% CI 81–93%) at 3 and 5 years, respectively. These findings were confirmed by a propensity score analysis accounting for selection bias.

**Conclusions:**

Trastuzumab plus chemotherapy should remain the preferred option in all patients with HER2-positive EBC with an indication for adjuvant treatment. However, a limited proportion of patients will need an alternative treatment approach, either because of contraindications or the patient’s preference. In these selected patients, trastuzumab monotherapy, eventually combined with endocrine agents, might be a reasonable option offering favorable long-term outcomes by addressing the high-risk profile associated with HER2-positive disease.

## Background

For over a decade, the monoclonal antibody trastuzumab has been the cornerstone of adjuvant treatment for HER2-positive early breast cancer (EBC) [1, 2]. Based on results from four large randomized trials [3–6], combined treatment with trastuzumab and chemotherapy (either as primary systemic or adjuvant treatment) is considered the standard of care in patients with this biologically aggressive subtype of breast cancer.

Although this evidence has led to unequivocal improvements in outcomes for the vast majority of patients with HER2-positive disease, the question remains as to whether there is a place for anti-HER2 therapy without chemotherapy in individually selected patients with EBC [7]. One major reason for this uncertainty is the fact that particular patient subgroups were underrepresented in the pivotal trials, including elderly patients [8, 9], those with significant concurrent disease, and those with small or low-risk tumors. In the latter subgroup, HER2-targeted therapy seems to be principally indicated, as several retrospective studies have shown that HER2 positivity leads to an unfavorable prognosis in patients with small cancers that are otherwise considered low risk [7, 10–13]. However, in view of the generally low rate of relapse events in these patients, chemotherapy-induced toxicity remains a major concern, leading to the question as to whether trastuzumab monotherapy is an adequate alternative option [7]. Hence, the issue of adjuvant trastuzumab monotherapy has repeatedly been discussed at international consensus meetings, resulting in weak recommendations and the recurrent request for randomized clinical trials. Unfortunately, such trials are difficult to perform due to the limited cohort size and the predictably low event rate. We therefore decided to approach this question within the framework of our database of about 4000 patients with EBC receiving trastuzumab.

This observational study [14] was started immediately after marketing authorization was received for Herceptin™ (trastuzumab) treatment in EBC. Its purpose was to obtain real-world evidence on routine usage of trastuzumab in Germany. As this was a non-interventional study with no criteria concerning patient inclusion or treatment (apart from trastuzumab), the database included patients who were receiving trastuzumab without any cytotoxic treatment. This offered the opportunity to analyze outcomes in this subgroup and compare them with patients treated according to the standard approach, both by crude comparison and by application of a propensity score method to account for the assumed presence of selection bias.

## Methods

### Patient population and methods of observation

Details of the organizational and legal framework of this non-interventional study (Roche ML20315), the selection criteria for inclusion in the observation procedure, and the scope of the documentation have been described previously [14]. In general, patients were treated and their disease course was assessed according to routine practice at the treating institution. Findings were prospectively documented on standardized case report forms. Data on treatment toxicity were mainly collected throughout the duration of adjuvant therapy, i.e. up to 12 months. The study started in 2006 and database lock for the analyses presented here was October 2013.

### Endpoint evaluation and statistical analyses

Relapse-free survival (RFS) and overall survival (OS) were calculated as the time between the baseline assessment before the first trastuzumab administration and the respective event. Surviving patients (without relapse for RFS) were censored at the last valid observation point. Event-related endpoints were analyzed using Kaplan-Meier methodology, with 95% confidence intervals (CIs) for event-free proportions at specific time points. Univariate analyses comparing the treatment subgroups were performed using the log-rank test, while hazard ratios (HRs) with 95% CIs were derived from Cox proportional hazards models [15]. In order to analyze the association between patient characteristics and the decision to withhold chemotherapy, *t* tests, Fisher’s exact tests, or appropriate trend tests for ordered categories were applied. All factors with an associated *P*-value <0.1 in univariate analysis were included in a multivariable logistic regression model.

Propensity score analysis [16], adjusting for selection bias when comparing the treatment subgroups with respect to RFS, was performed using the following prospectively planned steps: (1) covariate selection; (2) assessment of covariate balance before matching; (3) estimation of propensity scores by fitting a logistic regression model and matching procedure with a chosen sample size ratio of 1:1; (4) assessment of covariate balance after propensity score matching; and (5) estimation of the treatment effect with a log-rank test, stratified by matched pairs, with RFS as the primary endpoint. As sensitivity analyses, unstratified methods were also applied because accounting for matching in time-to-event endpoints remains controversial [17, 18]. The R statistical software (R Foundation for Statistical Computing; https://www.r-project.org; Version 3.0) and its “match-it” package were used.

All statistical analyses were of an exploratory nature, with *P ≤* 0.05 termed significant, without any adjustments for multiplicity applied. All reported *P*-values are two-sided.

## Results

### Baseline and treatment characteristics of patients with and without chemotherapy

Between September 2006 and July 2011, a total of 3940 eligible patients with HER2-positive breast cancer were recruited, 3935 of whom could be unequivocally categorized into groups with (*n* = 3703; 94%) or without (*n* = 232; 6%) any sequential or concurrent (neo)adjuvant chemotherapy. Patient and tumor characteristics are shown in Table 1.

**Table 1:** Patient and tumor characteristics

Characteristic	Chemotherapy	No chemotherapy	*P*-value
	(*n* = 3703)	(*n* = 232)	
Age, years			
Mean (range)	55.6 (20–100)	58.3 (27–87)	0.0008
< 60, *n* (%)	2298 (62)	128 (55)	
60– 69, *n* (%)	971 (26)	58 (25)	
≥ 70, *n* (%)	461 (12)	46 (20)	
ECOG performance status, *n* (%)			
0	2301 (63)	108 (47)	<0.0001
1	1298 (35)	113 (50)	
2–4	61 (2)	7 (3)	
Primary tumor stage, *n* (%)			
pT1/is	1750 (50)	114 (51)	0.73
pT2-4	1776 (50)	110 (49)	
Lymph node stage, *n* (%)			
pN0	1936 (52)	126 (54)	
pN1	1003 (27)	53 (23)	
pN2	411 (11)	28 (12)	
pN3	259 (7)	13 (6)	
NX	82 (2)	12 (5)	
No. of nodes involved, mean ± SD	2.2 ± 4.7	2.3 ± 5.1	0.63
Grading, *n* (%)			
Grade 1/2	1731 (47)	123 (55)	0.028
Grade 3	1927 (53)	101 (45)	
Hormone-receptor status, *n* (%)			
ER positive	2217 (60)	145 (62)	
PgR positive	1857 (50)	133 (57)	
Either ER or PgR positive	2332 (63)	156 (67)	0.20
Additional adjuvant treatment, *n* (%)			
Endocrine therapy	2079 (56)	131 (56)	0.99
Radiotherapy	2897 (78)	146 (63)	<0.0001

Total patient numbers may deviate from *n* = 3935 because of missing values for some characteristics

*ECOG* Eastern Cooperative Oncology Group, *ER* estrogen receptor, *PgR* progesterone receptor, *SD* standard deviation

Patients receiving no chemotherapy were almost 3 years older on average (*P =* 0.0008) and more often presented with worse performance status (*P* < 0.0001) than those who received chemotherapy. In contrast, tumor-related characteristics such as TNM staging or hormone receptor status (*P =* 0.20) differed only marginally between the cohorts. Only poorly differentiated histology showed a moderate association with administration of the more aggressive therapy approach (*P =* 0.028).

The administration of additional adjuvant endocrine treatment was equally common in both patient groups, but radiotherapy was more often omitted in patients who did not receive chemotherapy (*P* < 0.0001). No differences were detected between patients with and without chemotherapy with respect to trastuzumab exposure, with mean initial doses of 7.1 and 7.2 mg/kg body weight, mean number of cycles of 18.4 and 17.9, and mean duration of antibody therapy of 50.5 and 50 weeks, respectively.

### Multivariable analysis of treatment decision

The significant parameters in the univariate analysis were included in a logistic regression model with the chosen treatment category as the dependent variable; all retained their independent level of association (Table 2). There is an obvious strong correlation between the decision to treat a patient with chemotherapy and the decision to use radiotherapy. Therefore a second regression analysis was done using a model that excluded the radiotherapy factor; it yielded almost unchanged results for the other factors. For the same reason, irradiation was not included in the propensity score procedure (see below).

**Table 2:** Multivariable regression analysis of factors associated with treatment category

Factor^a^	Odds ratio^b^ [*P*-value]	
	Multivariable model 1^c^	Multivariable model 2^d^
Age: <65 vs ≥65 years	1.51 [*P* = 0.0056]	1.57 [*P* = 0.0020]
ECOG performance: 0 vs 1–4	1.80 [*P* = 0.00003]	1.84 [*P* = 0.00001]
Grading: Grade 1/2 vs grade 3	0.77 [*P* = 0.062]	0.77 [*P* = 0.058]
Radiotherapy: no vs yes	0.53 [*P* = 0.00002]	–

ECOG, Eastern Cooperative Oncology Group

^a^First group mentioned is reference; − = not in model. ^b^A value >1.0 indicates a higher probability of receiving Herceptin treatment without chemotherapy, as compared to reference group. ^c^Including radiotherapy in the analysis. ^d^Excluding radiotherapy from the analysis

### Trastuzumab-related toxicity

Among the patients receiving chemotherapy, adverse reactions related to cardiac function (all severity grades) were reported in 154/3703 cases (4.2%), with 93 (2.5%) assessed as grade 2–4 (Common Terminology Criteria for Adverse Events V.3). The corresponding numbers in the cohort receiving no chemotherapy were 5/232 (2.2%) and 4/232 (1.7%), respectively. The incidence of a pathological cardiac status during the baseline visit (detected by any type of cardiac monitoring) was similar between the two groups (7% and 6% in those receiving and not receiving chemotherapy, respectively). At the end of adjuvant treatment the proportion was 8% in both groups. However, the general recommendations for heart function assessment were not followed in a considerable number of patients. The rate of patients having echocardiography was only around 60% per three-month time interval [14]. Other presumed adverse drug reactions of severity grade 3/4 were rare in the monotherapy group: two cases of cardiac arrhythmia, two cases of dyspnea or other lung toxicity, and one patient with elevated liver enzymes.

### Long-term outcome: Crude analysis

**Fig. 1:**
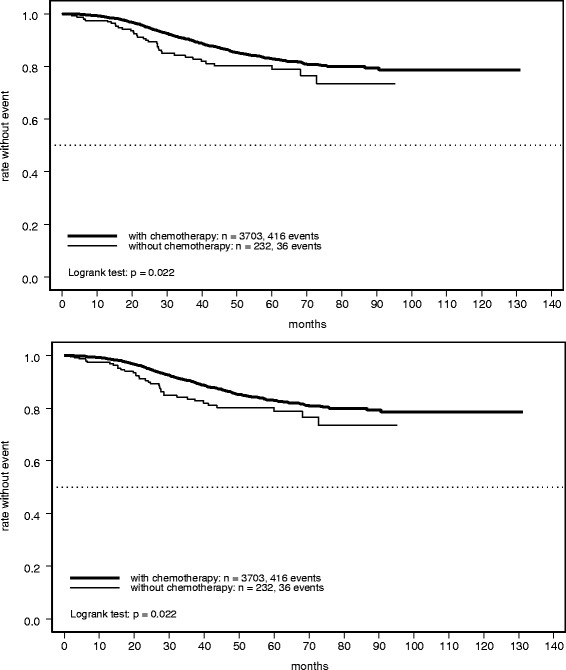
Kaplan-Meier plots of (**a**) relapse-free survival and (**b**) overall survival in patients with early breast cancer receiving adjuvant trastuzumab with or without chemotherapy

A total of 452 relapse-free survival events were observed before the database lock. In the chemotherapy group, the RFS rate was 90% (95% CI 89–92%) at 3 years and 83% (95% CI 81–85%) at 5 years. The corresponding rates were distinctly lower in the cohort receiving no chemotherapy: 84% (95% CI 78–89%) and 80% (95% CI 74–87%), respectively (Fig. 1a). The difference between treatment groups was statistically significant: HR 1.49 (95% CI 1.06–2.09; *P =* 0.022). A similar pattern was observed for OS, although with only marginally non-significant inferiority for the group receiving no chemotherapy, based on a total of 248 reported deaths (HR 1.56; 95% CI 1.00–2.44; *P =* 0.052) (Fig. 1b). The 3- and 5-year OS rates were 96% (95% CI 96–97%) and 90% (95% CI 89–92%) with chemotherapy, and 93% (95% CI 88–97%) and 87% (95% CI 81–93%) without chemotherapy, respectively.

### Long-term outcome: Propensity score analysis

Owing to the very high number of patients receiving chemotherapy, 204 monotherapy patients with a complete set of covariates available could be matched with 204 control patients, achieving perfectly balanced distributions for age (≥65 years: 35%), T stage (pT2–4: 51%), N stage (pN+: 43%), grading (grade 3: 46%), hormone receptor status (positive: 68%), and performance status (ECOG 0: 48%). As was expected, these proportions were very close to those described for the entire cohort without chemotherapy (Table 1).

**Fig. 2:**
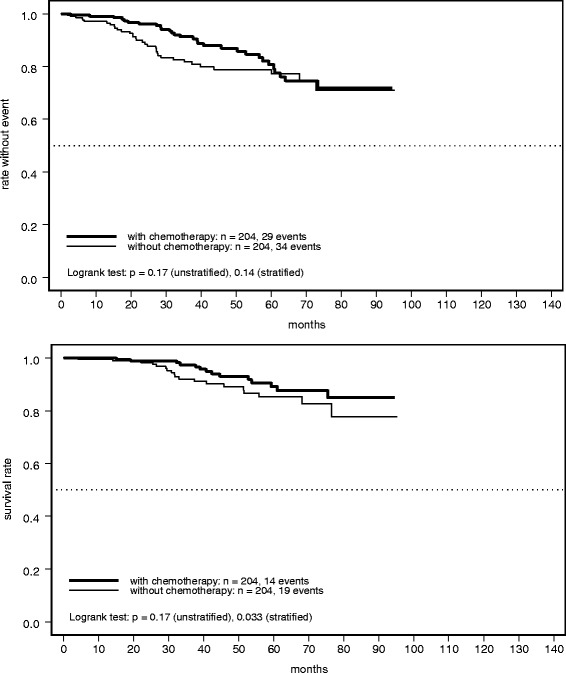
Kaplan-Meier plots of (**a**) relapse-free survival and (**b**) overall survival in propensity score matched patients with early breast cancer receiving adjuvant trastuzumab with or without chemotherapy

Figure 2a shows the RFS results for the matched samples, with an HR of 1.41 (95% CI 0.86–2.31; *P =* 0.17) in the unstratified analysis, and HR 1.49 (95% CI 0.88–2.52; *P =* 0.14) after stratification of the matched pairs. For OS, the corresponding results were HR 1.61 (95% CI 0.81-3.22; *P =* 0.17) and HR 2.35 (95% CI 1.05–5.25; *P =* 0.033) (Fig. 2b). The Kaplan-Meier curves and HRs did not differ qualitatively from the results of the crude analysis, suggesting that the superiority of the combined treatment is not an artifact caused by patient-selection bias. The wider CIs and larger *P*-values are an inevitable consequence of the limited number of observed events that remain after the matching procedure.

## Discussion

In the initial treatment of patients with HER2-positive breast cancer, administration of a specific agent targeting this epitope is almost universally indicated, independent from tumor stage and patient-defined characteristics, such as age. Focusing on the volume of available evidence, current treatment guidelines almost exclusively recommend the use of combination regimens of HER2-targeted therapy with either chemotherapy or a second targeted drug [2]. However, the efficacy of trastuzumab monotherapy was proven early in its clinical development program [7], even in heavily pretreated patients [19, 20]. (Likewise, the addition of trastuzumab to endocrine agents in patients with hormone receptor-positive disease was shown to be beneficial [21].) The use of trastuzumab monotherapy in the adjuvant setting, which is frequently mentioned as an alternative option, can only rely on analogy to this evidence derived from the advanced or neo-adjuvant breast cancer setting [7].

In order to analyze the actual prevalence of this treatment approach in routine clinical practice and to gain insight into its clinical efficacy, we screened the database of our German observational study and found a small but considerable subgroup of patients who received trastuzumab without chemotherapy. This contrasts to other real-life studies from the Netherlands and UK, which reported no or minimal numbers of such cases [22, 23]. Our data show that the decision to take this treatment approach is clearly associated with expected characteristics, such as older age or worse general health status, and less aggressive histology, rather than tumor stage. These results from real-world data reflect findings for hypothetical cases presented to US oncologists in a survey asking for treatment recommendations in older adults with HER2-positive EBC [24].

Although these characteristics show distinct and statistically significant trends of selection, they do not reflect a clearly defined subpopulation of our total cohort. One major limitation of our study is that our documentation did not include any information on the individual reasons for not administering chemotherapy. Obtaining this information was prohibited for legal reasons, as including this question on the record form would have implied use of a treatment option that did not comply with the trastuzumab marketing authorization, which is not allowed for this type of observational study in Germany.

Nevertheless, we assume that a large proportion of our trastuzumab monotherapy cohort consists of patients who refused to be treated with cytotoxic chemotherapy. Publications on this topic are sparse [25], but in an American interview-based regional survey of 119 women who did not receive guideline-recommended adjuvant therapy, patient refusal was the reported reason for 31% [26].

Long-term outcome after trastuzumab therapy without chemotherapy proved to be in an acceptable range, with an RFS rate of 80% after 5 years. However, in univariate comparisons against the cohort receiving chemotherapy, an advantage for the more aggressive approach was detected for both RFS and OS. We accounted for the presumed presence of selection bias by using a propensity score matching technique. This approach led to efficacy results (as reflected by HRs) that were comparable with the findings of the univariate analysis.

To gain an indication of the utility of trastuzumab monotherapy, we can compare our results against historical series of patients with HER2-positive breast cancer who did not receive adjuvant chemotherapy or trastuzumab. For example, a large cohort of 965 patients with small breast cancer tumors (T1a/bN0M0) from the MD Anderson Center had a 5-year RFS rate of only 77% [12]. Indirect comparisons (with the usual caveats) against our RFS estimate of 80% suggest considerable benefit from trastuzumab monotherapy. This benefit becomes more obvious when considering that in our cohort approximately 50% were T stage ≥2 and 41% were node positive, while proportions of hormone receptor positivity and endocrine treatment were roughly similar between the two studies.

## Conclusions

In conclusion, trastuzumab plus chemotherapy should remain the preferred option in all patients with HER2-positive EBC and an indication for adjuvant treatment. However, a limited proportion of patients will need an alternative treatment strategy, either because of contraindications or patient preference. In these selected patients, trastuzumab monotherapy, eventually combined with endocrine therapy, might be a reasonable option offering favorable long-term outcomes by addressing the high-risk profile associated with HER2-positivity.

## Abbreviations

CI: Confidence interval
EBC: Early breast cancer
ECOG: Eastern Co-operative Oncology Group
ER: Estrogen receptor
HER2: Human epidermal growth factor receptor 2
HR: Hazard ratio
OS: Overall survival
PgR: Progesterone receptor
RFS: Relapse-free survival
SD: Standard deviation
